# Variation in Thickness of Embryo Covering Structures and Their Role in the Regulation of Seed Physiological Dormancy of *Chenopodium hircinum* (Amaranthaceae)

**DOI:** 10.3390/plants13192832

**Published:** 2024-10-09

**Authors:** Paola Fernanda Agüero-Martínez, Leonardo Cardozo, Carlos A. Gómez, Diego López-Spahr, Carol C. Baskin, Daniel Bertero, Guadalupe Galíndez, Ramiro Curti

**Affiliations:** 1 Facultad de Ciencias Naturales, Universidad Nacional de Salta (UNSa), Av. Bolivia 5150, Salta 4400, Argentina; fer_agumar1606@hotmail.com (P.F.A.-M.); lgcunsa14@gmail.com (L.C.); carlosunsa@gmail.com (C.A.G.); lopezspahr.diego@inta.gob.ar (D.L.-S.); rcurti@agro.uba.ar (R.C.); 2 Laboratorio de Microscopía Electrónica de Barrido (LASEM), Consejo Nacional de Investigaciones Científicas y Técnicas (CONICET), Universidad Nacional de Salta (UNSa), Av. Bolivia 5150, Salta 4400, Argentina; 3 Estación Experimental Agropecuaria Salta, Instituto Nacional de Tecnología Agropecuaria (INTA), Ruta Nac. 68, Km 172, Salta 4403, Argentina; 4 Department of Biology, University of Kentucky, Lexington, KY 40506-0225, USA; carol.baskin@uky.edu; 5 Department of Plant and Soil Sciences, University of Kentucky, Lexington, KY 40546-0321, USA; 6 Cátedra de Producción Vegetal e Instituto de Investigaciones Fisiológicas y Ecológicas Vinculadas a la Agricultura (IFEVA)—Consejo Nacional de Investigaciones Científicas y Técnicas (CONICET), Facultad de Agronomía, Universidad de Buenos Aires, Av. San Martín 4453, Buenos Aires 1417, Argentina; bertero@agro.uba.ar; 7 Centro Científico Tecnológico (CCT)-Salta-Jujuy, Consejo Nacional de Investigaciones Científicas y Técnicas (CONICET), Rivadavia 940, Salta 4400, Argentina

**Keywords:** breaking dormancy, *Chenopodium*, domestication, dormancy-breaking treatments, germination, intraspecific variability, morpho-anatomical traits, physiological dormancy, seed coat

## Abstract

*Chenopodium hircinum*, the putative wild ancestor of quinoa, is a source of traits that could improve the tolerance of crop quinoa to high temperatures. However, seeds of *C. hircinum* have physiological dormancy (PD), which is an obstacle for plant propagation and use in breeding programs. We studied the intraspecific variability in morpho-anatomical traits of embryo covering structures and their association with PD. We also evaluated the effects of different dormancy-breaking treatments on PD alleviation and germination. Seeds were dispersed with a remnant perianth and a persistent pericarp that could be removed by scraping. The seed coat was formed by palisade cells impregnated with tannins, and the seed contained a thin layer of peripheral endosperm surrounding the embryo. In our investigation, the thickness of the pericarp (P) and/or seed coat (SC) varied among populations. Populations with higher P and/or SC thickness showed lower percentages of germination and water absorption. The combined dormancy-breaking treatment (bleach + perforated coverings + gibberellic acid) promoted dormancy release and increased germination. *C. hircinum* seeds showed non-deep physiological dormancy. Based on previous knowledge about quinoa, and our results, we conclude that embryo coverings, especially the seed coat, have an important role in dormancy control, imposing a mechanical restraint on radicle emergence.

## 1. Introduction

The genus *Chenopodium* includes cultivated species of great economic importance in the Andean and Central American regions, one of which is *C. quinoa* (quinoa). This species is one of the most significant crops due to its nutritive value and tolerance of several abiotic stresses, including drought, salinity, and frost [1,2,3]. However, this crop has low yields in environments where temperatures exceed 35 °C [4]. *C. hircinum,* which is the putative wild ancestor of quinoa, is a weed that inhabits disturbed environments such as road edges and forests in primary succession, and it occupies warmer and harsher environmental niches than cultivated quinoa [5]. In the northwestern region of Argentina, the distribution of *C. hircinum* partially overlaps that of cultivated quinoa. Therefore, some of these populations could harbor traits (e.g., longer phenological cycle, lower stomatal conductance) that, if transferred to quinoa, could improve the crop’s adaptation to warm locations [4]. However, mature seeds of *C. hircinum*, show physiological dormancy (PD) at the time of dispersal [4], which is an obstacle for plant propagation and use in breeding programs.

Seed dormancy has been defined as the incapacity of seeds to germinate in a specified period of time under any combination of normal physical environmental factors that are otherwise favorable for its germination [6]. PD is the most prevalent and widespread class of seed dormancy in nature, affecting over 90% of the world’s species, including gymnosperms and major angiosperm clades [6,7]. Seeds with PD have water-permeable seed coats and fully developed embryos, and PD occurs due to an inherent physiological mechanisms in the embryo that limit its growth potential. Thus, the embryo does not have enough push power to overcome the mechanical resistance of its covering structures such as the endosperm, seed coat, and/or indehiscent fruit walls [7]. There are three levels of PD, and the most common one is non-deep PD, which is found in seeds of *Arabidopsis thaliana* and, thus, has received the most attention in terms of dormancy-break and germination requirements [7,8,9,10]. In general, dormancy is associated with high levels of abscisic acid (ABA) that decrease during dormancy-break, and germination is associated with an increase in gibberellic acid (GA) [7,10].

The exogenous application of GA, cold stratification (0–10 °C), and/or dry storage are some of the most common treatments that overcome PD, but the effective treatment varies with the species [6,7]. Thus, GA enhances the embryo’s growth potential, whereas cold stratification and dry storage reduce sensitivity to ABA and/or a decrease its concentration [7]. For example, in several Amaranthaceae species, including species of *Chenopodium* and *Amaranthus*, with non-deep PD, germination increases after short periods of cold stratification or gibberellin treatments [7]. On the other hand, seed-coat scarification (e.g., mechanically, chemically) can promote germination, mainly due to a release of the mechanical resistance of the covering structures on the embryo. However, after the embryo has gained full growth potential it can overcome the resistance of the covering structures [7].

In general, seeds of *C. quinoa* are not dormant [11]; however, Ceccato et al. [12] reported that two cultivars of quinoa, Chadmo and 2-Want, have PD regulated mainly by the seed coat. They reported a positive association between the thickness of the seed coat and the degree of PD and an increase in seed germination percentage after seed-coat perforation, which allowed the release of ABA into the medium. These authors also suggested that a reduction in dormancy imposed by the seed coat could be mediated by the influence of environmental conditions on coat thickness and/or other seed traits during seed development. On the other hand, for *C. hircinum,* Bruno and Whitehead [13] mentioned that its seeds have comparatively thicker seed coats than quinoa, and Curti et al. [5] reported that the combination of bleach, GA, and seed-coat perforation enhanced seed germination. However, seed traits, seed dormancy mechanisms, and how different treatments can break dormancy remain understudied for *C. hircinum*.

Our objective was to determine the intraspecific variability in morpho-anatomical traits of embryo covering structures and their association with PD in seeds of *C. hircinum*. Specifically, we (i) characterized seed morpho-anatomy traits; (ii) investigated the role of embryo covering structures, specifically the pericarp and seed coat, in the regulation of PD; (iii) evaluated the effects of different dormancy-breaking treatments on PD alleviation and germination; and (iv) determined intraspecific variability of all these characteristics. We expected that: (i) embryo covering structures would regulate PD mainly through mechanical resistance to radicle emergence, i.e., seeds with thicker coverings would show low or no initial germination, in contrast with seeds with thinner coverings; (ii) dormancy-breaking treatments that included perforation of the embryo covering structures would alleviate seed dormancy and increase seed germination; and (iii) seed dormancy would differ among populations, and these differences would be associated with differences in thickness of embryo covering structures.

## 2. Results

### 2.1. Seed Traits

**Figure 1:**
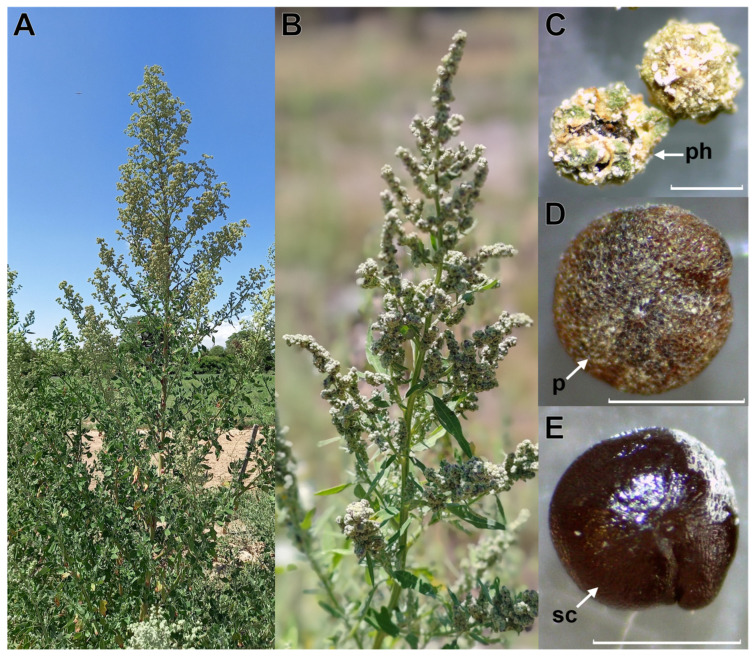
Plant of *Chenopodium hircinum* (**A**), inflorescence (**B**), seeds with perianth (**C**), seeds with pericarp (**D**), and seeds with seed coat (**E**). Abbreviations: p, pericarp; ph, perianth; sc: seed coat. Scale bars in (**C**–**E**) = 1 mm.

**Figure 2:**
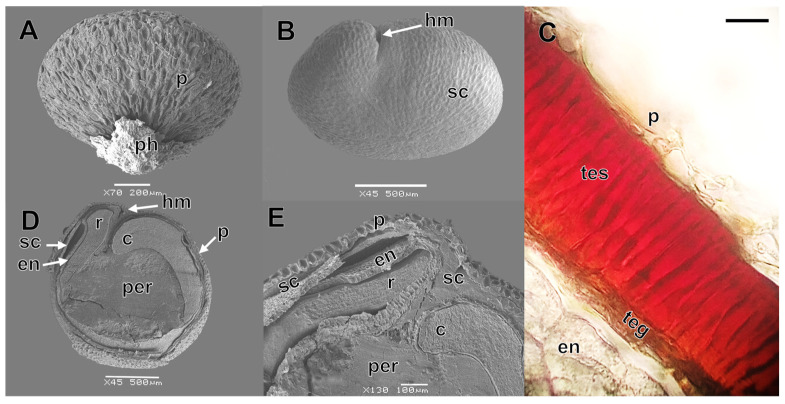
Scanning electron micrographs of the fruit (**A**), seed (**B**) and cross sections of the seed (**D**,**E**) and under an optical microscope (**C**) of *Chenopodium hircinum*. Abbreviations: c, cotyledon; en, endosperm; hm, hilar–micropylar region; per, perisperm; p, pericarp; ph, perianth; r, radicle; sc, seed coat; teg, tegmen; tes, testa. Scale bar in (**C**) = 20 μm.

For all populations at Cafayate (C1, C2, and C3) and San Carlos (S1 and S2) localities, dispersed seeds were covered with a dry remnant perianth and a persistent pericarp that was readily removed by scraping (Figure 1C,D and Figure 2A). The pericarp was dry, reticulate, and light brown in color, and consisted of one undifferentiated layer of parenchymatous cells (Figure 1D and Figure 2A,C). The seed coat was dark brown to black, reticulate–alveolate and consisted of two layers: testa and tegmen (Figure 1E and Figure 2B–E). The testa was formed by one layer of large palisade cells impregnated with tannin-like substances, while the tegmen was formed by one thin layer of flattened, apparently collapsed cells (Figure 2C). Internally, a peripheral curved embryo was observed around a large central perisperm, and the radicle and cotyledons were oriented towards the hilar–micropylar region (Figure 2D,E). The endosperm covered the entire embryo, formed by six to seven cell layers in the hilar–micropylar region and only one cell layer in the rest of the seed. Saponins were found only in the pericarp, and they exhibited a red-brown color when treated with sulfuric acid.

Seed mass ranged between 13.63 and 25.45 mg and varied among populations, with seeds from S1 and C2 showing the highest and the lowest values, respectively (Table 1). Both the tetrazolium test and cut test indicated high seed viability (≥90%) in all populations, and no significant differences were registered among them. However, the initial germination percentage varied significantly among populations (Table 1). Thus, except for seeds from S1 that germinated >50% with a t_50_ of 3 ± 1 d, seeds from the other populations germinated ≤17% and, therefore, t_50_ could not be estimated.

### 2.2. Seed Dormancy and Germination

Thickness of pericarp, seed coat, and both embryo covering structures combined (ECSs), and the ECSs thickness:seed mass ratio varied among populations (Table 1). Thus, seeds from S2 and C1 showed the highest values for pericarp and seed-coat thickness, respectively, and both showed higher embryo covering structure thickness than the other populations. The ECSs thickness:seed mass ratio was significantly higher in C1, C2, and S2 than in C3 and S1, whereas micropylar endosperm thickness did not vary among populations (Table 1).

**Figure 3:**
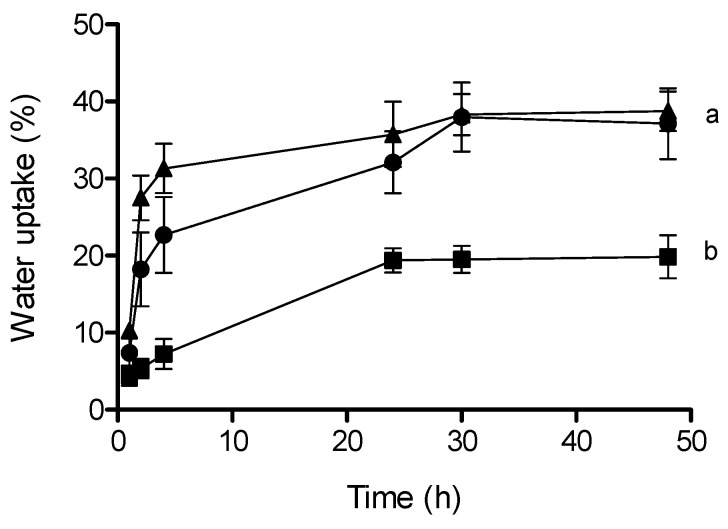
Cumulative water uptake (%) for each treatment: Seeds without pericarp (■), intact seeds (●), and perforated seeds (▲). Values with different letters indicate significant differences between treatments included in the analyses (DGC test *p* < 0.05).

Water uptake (%) also varied among populations (Table 1). After 12 h of imbibition, the mass of intact seeds from S1 increased by around 26%, and after 24 h, around 48%. In contrast, after 24 h, seeds of the other populations increased by ≤28% of their initial seed mass. No germination was registered during the experiment for all populations. In addition, water uptake was affected significantly by treatment, but it was not affected either by population or by the treatment × population interaction (Figure 3). Thus, water uptake was not different between intact seeds and perforated seeds, but, in both of these, it was significantly higher than in seeds without pericarp.

Seed mass correlated negatively with seed-coat thickness and positively with pericarp thickness, water uptake percentage, and germination percentage of intact seeds. In addition, the thickness of seed coat and embryo covering structures correlated negatively with percentage of water uptake and with germination percentage of intact seeds (Table 2).

**Figure 4:**
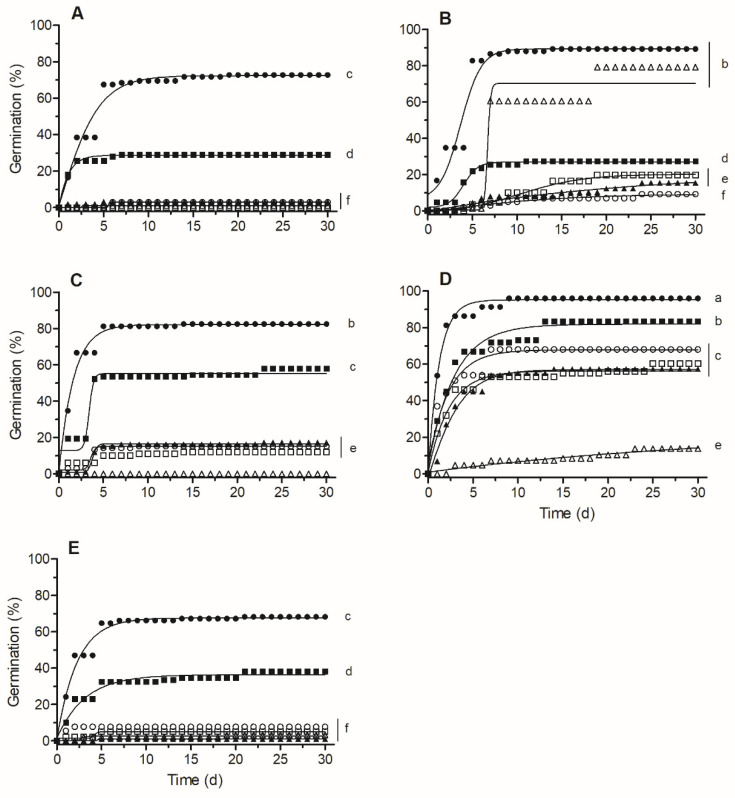
Cumulative germination (%) for each treatment: bleach (○), GA (□), perforated (■), combined (●), stratification (∆), and control (▲); and population: C1 (**A**), C2 (**B**), C3 (**C**), S1 (**D**), and S2 (**E**). The germination time course curves were calculated with non-linear adjustment to the Gompertz equation. Values with different letters indicate significant differences between populations and treatments included in the analyses (DGC test *p* < 0.05).

Germination percentage and t_50_ were affected significantly by population, treatment, and by interaction (Figure 4). Thus, the highest percentage and lowest t_50_ were registered in the combined treatment of S1 (96% and <1 d, respectively), followed by the combined treatment of C2 and C3 and perforated treatment of S1 (on average 87% and 2–3 d). Stratification treatment had a significant effect on germination only in seeds from the C2 population (Figure 4). For C1 and C3, no germination was registered in the GA or stratification treatments.

## 3. Discussion

In agreement with previous studies of other *Chenopodium* species and related genera [12,14,15,16,17], seeds of *C. hircinum* showed the general structures of *Chenopodium* seeds (Figure 1 and Figure 2). Thus, seeds disperse with remnants of perianth and a persistent pericarp that can be easily removed by friction, successive water washes, and/or bleach application. The pericarp was formed by one layer of cells and the seed coat (or episperm) consisted of the testa (or exotesta) and the tegmen (or endotegmen). An embryo wrapped the perisperm (storage tissue) and a thicker endosperm was present in the micropylar region than in other parts of the seed. However, we also found two seed traits that are not present in *C. quinoa* seeds [12,16]: (1) a palisade layer impregnated with tannin-like substances in the testa, which gave the seed coat a dark color and (2) a continuous peripheral endosperm formed by one or two layers surrounding all the embryo. Toderich et al. [3] also reported the presence of palisade layers with tannins in *C. quinoa* seeds, but only in those lines with dark seed coats, and suggested that it is involved in seed-coat thickness. On the other hand, López-Fernández and Maldonado [18] and Burrieza et al. [14] mentioned that during seed development of *C. quinoa*, the embryo grows at the expense of the chalazal and peripheral endosperms which are progressively dismantled, whereas micropylar endosperm, that stores lipids and proteins, is used by the embryo during germination. The presence of a dark seed coat and a peripheral endosperm are likely ancient traits that would have been lost during the processes of *C. quinoa* domestication [3,17]. Further histological studies could help to understand the origin and composition of these tissues and their functions in seed dormancy and germination.

Seeds of all populations dispersed with a high initial viability but a low capacity to germinate, indicating the presence of PD at seed maturity (Table 1). Similar results have been reported for *C. hircinum* [5] and *C. quinoa* [12] and for other *Chenopodium* species [7,19]. In addition, seed mass and initial germination exhibited variation among populations. Thus, except for S1, which showed higher seed mass and germination percentage, the other populations showed lower seed mass and germination (Table 2). Several studies have indicated that small seeds are more likely to show dormancy and low germination percentages and rates than large seeds [7,20].

These differences in seed mass were also accompanied by differences in the thickness of seed covering structures (i.e., pericarp and/or seed coat), with smaller seeds showing thicker protective coverings than larger seeds (Table 1 and Table 2). Thickness values, on average, for all populations were similar to those reported by Sukhorukov and Zhang [17] for other *Chenopodium* species (i.e., <40 μm for pericarp and between 15–50 μm for seed coat), but seed coats values were higher than those reported for *C. quinoa* (<20 μm) [12,13,17].

As we expected, populations with thicker coverings (C1 and S2) showed low or no initial germination, in contrast with seeds with thinner coverings (S1; Table 2). In the same sense, Ceccato et al. [12] suggested that the level of dormancy in cultivars of *C. quinoa* would be associated with seed-coat thickness and that dormancy imposed by the embryo would be expressed under environmental conditions where dormancy imposed by the seed coats is reduced. This seems to be the case in the S1 population, which exhibited seeds with higher seed mass and thinner seed coverings, probably due to higher water availability, since it has higher annual precipitation that can be invested in production of larger seeds, with large embryos, thin coats, and lower levels of seed dormancy [7,11,21,22]. On the other hand, Curti and Bertero [23] mentioned that changes in seed size did not allow one to distinguish between cultivated and wild forms, but changes in seed-coat thickness did distinguish ancient forms, as we confirm for *C. hircinum*, from domesticated *C. quinoa*, with lower values in domesticated species. One reason for human selection of seeds with thin coats is that they are easier to prepare to consume than seeds with thicker coats, as is the case for *C. quinoa* [7].

In seeds with physiological dormancy, seed coverings control germination mainly by preventing radicle protrusion [7]. As we expected, seed coverings regulated dormancy of *C. hircinum* seeds, mainly through mechanical resistance to radicle emergence, since only after perforation of seed coverings did germination percentages increase in all populations (Figure 4). On the other hand, both intact seeds and perforated seeds absorbed water (which is one of the characteristics of PD, i.e., permeable seed coats; Figure 3). In this sense, Sukhorukov et al. [17] indicated that cells of dry fruits of many *Chenopodium* species regain turgor pressure after soaking in water, using the increased water availability for rapid hydration, contributing to the recovery of seed turgor pressure. Additionally, the presence of saponins in the pericarp facilitate seed germination by shortening the lag phase during seed imbibition and weakening the seed coat, particularly under saline stress conditions [24]. In contrast, in seeds without pericarp (i.e., only with seed coat) the rate of water absorbed was lower than in intact and perforated seeds (Figure 3), indicating that seed coat also helped regulate water uptake. It has been reported that the presence of a thick seed coat formed by a palisade layer would reduce seed-coat permeability [9,25] and that tannins inhibit seed germination [3,16].

Finally, the best method for breaking dormancy in all populations was the combination of bleach, perforation of seed coverings (scarification), and exogenous application of GA (Figure 4). Bleach can act as a chemical scarifier because it can oxidize inhibitors such as tannins, reducing mechanical resistance of the pericarp and seed coat, and increasing water absorption [26]. Seed perforation decreased mechanical resistance to radicle emergence and allowed an increased rate of water absorption, and it can also allow for the release of ABA, as has been reported for *C. quinoa* [12]. The exogenous application of GA enhances embryo growth potential, which promotes seed germination [7]. However, we found intraspecific variability in the response to the different breaking-dormancy treatments evaluated, with some populations (e.g., S1 and C2) more sensitive to stratification and GA than the other populations. According to these results, *C. hircinum* seeds show non-deep physiological dormancy [6,7], as has been also reported for other *Chenopodium* species [19].

The knowledge of seed traits and seed dormancy regulation in *C. hircinum* provides useful information for developing protocols to break dormancy and increase seed germination for plant propagation and use in breeding programs. In addition, the intraspecific variability in seed mass, seed covering structure thickness, and dormancy-breaking treatments should be considered before recommending the use of a specific seed source for these programs. Finally, these results increase our knowledge about quinoa’s wild ancestor and the domestication process.

## 4. Materials and Methods

### 4.1. Study Species and Seed Collection

*Chenopodium hircinum* Schrad. is an annual herbaceous species with erect stems that can reach up to 2 m in height (Figure 1). The flowers are clustered in dense panicles of glomerules. The fruits (utricles, commonly called achenes) are covered by remnants of the sepals (perianth) and a dry farinose pericarp. The seeds are perispermous and 1.0 to 1.2 mm in diameter, and the seed coat is dark brown to black [27]. Plants flower between spring and summer (September and April), and seed maturation within the inflorescence is asynchronous [28].

In January 2022, numerous fruits (hereafter seeds) were collected from at least 15 individuals of five populations of Cafayate and San Carlos localities, in the province of Salta, Argentina (Table 3). These populations were selected from a total of 33 populations of *C. hircinum* across the entire distribution range of *C. hircinum* (latitude 25°48 0–35°25 0 S, longitude 59°21 0–64°58 0 W, and altitude 26.0–2.058 m a.s.l.) and represent those from warmer and drier environments [4]. They are of particular interest as they may exhibit traits of heat tolerance that could be beneficial for *C. quinoa* breeding programs. In the laboratory, the seeds were stored in paper bags at room temperature until used (<14 d).

### 4.2. Seed Traits

Immediately after seed collection, for each population, we determined: seed morpho-anatomical traits, seed mass, seed viability, and initial seed germination. Seed morpho-anatomical traits were determined for 40 fresh seeds. Ten seeds were observed and photographed under a stereoscopic microscope (Motic SMZ-171). Ten intact and ten longitudinally sectioned seeds also were fixed in FAA (formaldehyde–alcohol–acetic acid) for 48 h. After fixation, the seeds were dehydrated in ethanol, critical-point dried with CO_2_, coated with gold, and observed under a scanning electron microscope (SEM; JEOL JSM-6480 LV). Digital photographs of intact and sectioned seeds were taken, and they were processed with Image J v. 1.53k [29] to estimate thickness of the layers around the embryo. Finally, to characterize the anatomy of embryo covering structures, 10 fresh seeds were transversely sectioned in vivo with a sliding microtome (Leica SM 2000R) at 20 µm thickness, using fennel pith as support. Sections were mounted on a microscope slide with a water:glycerol solution 1:1 [30] and observed with an optical microscope (OM; Leica DM 2500). Additionally, the presence of saponins in the pericarp and seed coat was determined following Zarlavsky [31]. To determine if saponins were present, two lots of 50 intact seeds (i.e., with pericarp) and 50 seeds without pericarp were immersed in sulfuric acid (98%) for 30 min, and the color of the solution was recorded. A red to violet color would indicate the presence of saponins. Pericarp was removed by immersing the seeds in bleach (NaClO, 3.5%) for 10 min. Seed mass was determined by weighing four replicates of 25 seeds each with a precision balance to 0.0001 mg accuracy (Denver Instrument APX-200, Denver Instrument Company, Denver, CO, USA).

Seed viability was evaluated for four replicates of 25 seeds each for each population, using the tetrazolium chloride staining test [32]. Initial seed germination was determined for four replicates of 25 seeds each for each population. Seeds were sown in Petri dishes on two sheets of filter paper moistened with distilled water and incubated in a chamber at 20/30 °C, 12/12 h light/dark (optimal conditions) [5]. Germination (i.e., radicle emergence) was recorded daily for 30 d. At the end of the germination tests, when no additional germination had occurred for 2 weeks, a cut test was conducted to determine the viability of the non-germinated seeds (soft or firm, i.e., dead or viable, respectively). The number of viable seeds per replicate was the number of firm non-germinated seeds + the germinated seeds. The final germination percentage was calculated based on the total number of viable seeds.

Time–course cumulative germination curves obtained for the control and those receiving different treatments (see below) were used to estimate the time required for completion of 50% germination of viable seeds (t_50_). For this purpose, we fitted the time–course cumulative germination data to the Gompertz equation: Y = C × exp (exp (B × (X − M))), where Y is the percentage of germination, X is time in days, C is the maximum percentage of germination reached by the seed batches (the asymptote of the curve), B is the maximum germination rate standardized by the maximum percentage of germination, and M is the value of time, in days, corresponding to the inflection point of the curve (moment at which the maximum rate of germination is reached). Viable seeds that did not germinate after 30 days were considered dormant [7].

### 4.3. Role of Embryo Covering Structures in Dormancy Regulation and Seed Germination

The role of embryo covering structures of seeds (ECSs) in dormancy regulation was evaluated through ECSs thickness as an estimator of mechanical resistance to radicle emergence. Since micropillar endosperm thickness did not vary among populations (see results), the CS thickness was estimated through pericarp (P) + seed coat (SC) thickness, which was estimated by analyzing digital photographs of longitudinally sectioned seeds in the hilar–micropylar region. The ECSs thickness:seed mass (SM) ratio also was estimated.

Additionally, the role of embryo covering structures on water absorption was evaluated through imbibition curves [7] on (1) intact seeds, (2) seeds without pericarp, and (3) intact perforated seeds. Four replicates of 25 seeds from each population were initially weighed using a digital balance (0.0001 g precision) and then sown in Petri dishes imbibed with distilled water and incubated in a chamber at 20/30 °C, 12/12 h light/dark. Seeds were removed from the dishes at 1–2 h intervals for the first 8 h and then every 24 h, blotted dry with filter paper, and reweighed. The weighing procedure was continued until the mass of the seeds remained constant (ca., 48 h) or seeds germinated. Water uptake percentage was calculated as (final weight − initial weight)/initial weight × 100 [32]. With this data, we built the imbibition curve (i.e., cumulative water uptake vs. time) for each treatment. For the perforation treatment, seeds were punctured with a 60 mm histological needle in the perisperm area (to avoid embryo damage) [12].

### 4.4. Dormancy-Breaking Treatments

To determine the effect of bleach, GA, and seed covering perforation on breaking dormancy and seed germination [4], the following treatments were applied: (1) bleach: seeds were immersed in bleach (NaClO 3.5%) for 2 h, (2) GA: seeds were immersed in GA_3_ (1000 µL) for 24 h, (3) perforated, (4) combined: seeds were immersed in bleach, then perforated and, finally, immersed in GA, and (5) control: intact seeds. Given that seeds of Amaranthaceae species with PD respond to cold stratification [7], the effect of cold stratification on breaking dormancy was also evaluated by sowing seeds on filter paper soaked in distilled water and incubated in darkness at 5 °C for 4 weeks.

For all populations and treatments, 4 replicates of 25 seeds were used, and after each treatment (or control) seeds were then sown in Petri dishes on 2 sheets of filter paper moistened with distilled water and incubated in a chamber at 20/30 °C, 12/12 h light/dark. The number of germinated seeds and t_50_ were determined as previously described.

### 4.5. Statistical Analysis

Seed morpho-anatomical traits, seed mass, seed viability (%), water uptake (%), and t_50_ were compared among populations using ANOVA. For initial germination and dormancy-breaking treatment data, general linear mixed models for binomial distribution and logit link functions were used to explain variations in number of germinated seeds. As the number of viable seeds was not the same for all experimental units, this number was entered as a covariable. For all analyses, populations and treatments were considered as fixed-effect factors, and all factor interactions were included in the models. AIC and BIC information criteria were used to select the most parsimonious model, and the residual deviance/degrees of freedom ratio was calculated to assess if the goodness of fit of the model was reasonable and if there was no over-dispersion. The post hoc DGC test of multiple comparisons of means [33] was used to establish which mean differences, between levels of each factor or between their combinations, were significant (*p* < 0.05). We explored the correlation between seed mass, morpho-anatomical seed traits (pericarp, seed coat, and both embryo covering structures), and physiological variables (percentage of water uptake after 24 h of imbibition and initial germination percentage), evaluating the significance of the Pearson correlation coefficient (*p* < 0.05). All statistical analyses were performed with the InfoStat package [34].

## 5. Conclusions

We documented intraspecific variability in seed traits and the role of embryo covering structures in the regulation of PD in five populations of *C. hircinum* growing in the northwestern region of Argentina. *C. hircinum* seeds showed the general morpho-anatomical traits of *Chenopodium* genera; however, a testa with a palisade layer impregnated with tannins and a continuous peripheral endosperm surrounding all the embryo were also present. Both are considered primitive characteristics expected to be found in *C. hircinum*, the wild ancestor of *C. quinoa*, and that would be lost during domestication processes. Pericarp and/or seed-coat thickness varied among populations, and seeds with thicker coverings had a higher degree of physiological dormancy than those with thinner coverings. In particular, thickness of the seed coat regulated dormancy by imposing mechanical resistance to radicle emergence and also decreasing water absorption. The combination of bleach, covering perforation, and GA was the best method for breaking seed dormancy, which corroborates the presence of non-deep PD and the role of embryo covering structures in seed dormancy. All these results extend our knowledge about the natural regeneration of *C. hircinum* in the different environments where it grows and the phenotypic variability in populations that should be considered before recommending the use of a specific seed source for plant propagation and breeding programs.

## Figures and Tables

**Table 1:** Mean (± standard error) of initial viability, germination, seed mass (SM), morpho-anatomical seed traits: pericarp (P), seed coat (SC), endosperm (En), embryo covering structures (ECSs = P + SC), embryo covering structure thickness:seed mass ratio (ECSs:SM); and water uptake (% initial mass basis) after 12 and 24 h of imbibition for each population. Values with different letters indicate significant differences among populations (DGC test *p* < 0.05).

Population	Viability (%)	Germination (%)	SM (mg)	P (μm)	SC (μm)	En (μm)	ECSs (μm)	ECSs:SM	Water Uptake (%)	
									12 h	24 h
C1	96 ± 2 ^a^	3 ± 3 ^c^	17.35 ± 0.24 ^c^	23.10 ± 0.15 ^c^	49.85 ± 6.04 ^a^	30.34 ± 0.69 ^a^	68.17 ± 5.11 ^a^	3.90 ± 0.31 ^a^	11.28 ± 1.19 ^c^	17.15 ± 1.05 ^c^
C2	90 ± 3 ^a^	16 ± 4 ^b^	13.63 ± 0.71 ^d^	19.40 ± 0.36 ^d^	32.71 ± 5.01 ^b^	30.09 ± 0.47 ^a^	49.63 ± 6.53 ^b^	3.65 ± 0.73 ^a^	17.37 ± 0.92 ^b^	25.38 ± 2.91 ^b^
C3	93 ± 2 ^a^	17 ± 2 ^b^	21.25 ± 0.62 ^b^	22.43 ± 0.42 ^c^	30.90 ± 2.05 ^b^	30.15 ± 0.49 ^a^	52.67 ± 2.72 ^b^	2.44 ± 0.11 ^b^	19.98 ± 1.22 ^b^	27.98 ± 2.89 ^b^
S1	98 ± 1 ^a^	57 ± 9 ^a^	25.45 ± 0.45 ^a^	26.60 ± 1.78 ^b^	23.27 ± 1.30 ^b^	29.81 ± 0.59 ^a^	48.66 ± 1.82 ^b^	1.93 ± 0.11 ^b^	25.82 ± 0.78 ^a^	48.08 ± 3.18 ^a^
S2	94 ± 2 ^a^	1 ± 1 ^c^	20.90 ± 0.38 ^b^	32.03 ± 0.71 ^a^	34.03 ± 4.76 ^b^	31.09 ± 0.54 ^a^	66.87 ± 5.99 ^a^	3.20 ± 0.32 ^a^	10.52 ± 0.58 ^c^	16.27 ± 0.80 ^c^

**Table 2:** Coefficient values (r) of the correlations between seed mass (SM); morpho-anatomical seed traits: pericarp (P), seed coat (SC), and embryo covering structures (ECSs); and physiological variables: percentage of water uptake after 24 h of imbibition (%WA) and initial germination percentage (%G). Bold values indicate the significance of the lineal correlation at *p* < 0.05.

	SM	P	SC	ECSs	%WA
P	**0.53**				
SC	**−0.46**	−0.05			
ECSs	−0.17	0.36	**0.91**		
%WA	**0.59**	−0.07	**−0.51**	**−0.59**	
%G	**0.59**	−0.05	**−0.52**	**−0.58**	**0.94**

**Table 3:** Geographic (latitude, longitude, and altitude) and climatic (annual mean temperature and annual precipitation) data for each population and locality of seed collection. Climatic data were extracted from Worldclim (https://www.worldclim.org/data/worldclim21.html; accessed on 1 September 2023).

Locality	Population	Latitude (S)	Longitude (O)	Altitude (m.s.m)	Temperature (°C)	Precipitation (mm)
Cafayate	C1	26.06	65.97	1635	17.06	160
	C2	26.06	65.96	1618	17.13	162
	C3	26.09	65.97	1612	17.04	171
San Carlos	S1	25.88	65.93	1634	17.25	175
	S2	25.90	65.93	1623	17.27	159

## Data Availability

Due to privacy issues, the data presented in this study are available on request from the corresponding author.
